# A variant of the *Escherichia coli* anaerobic transcription factor FNR exhibiting diminished promoter activation function enhances ionizing radiation resistance

**DOI:** 10.1371/journal.pone.0199482

**Published:** 2019-01-23

**Authors:** Steven T. Bruckbauer, Joseph D. Trimarco, Camille Henry, Elizabeth A. Wood, John R. Battista, Michael M. Cox

**Affiliations:** 1 Department of Biochemistry, University of Wisconsin – Madison, Madison, Wisconsin, United States of America; 2 Department of Biological Sciences, Louisiana State University and A & M College, Baton Rouge, Louisiana, United States of America; Saint Louis University, UNITED STATES

## Abstract

We have previously generated four replicate populations of ionizing radiation (IR)-resistant *Escherichia coli* though directed evolution. Sequencing of isolates from these populations revealed that mutations affecting DNA repair (through DNA double-strand break repair and replication restart), ROS amelioration, and cell wall metabolism were prominent. Three mutations involved in DNA repair explained the IR resistance phenotype in one population, and similar DNA repair mutations were prominent in two others. The remaining population, IR-3-20, had no mutations in the key DNA repair proteins, suggesting that it had taken a different evolutionary path to IR resistance. Here, we present evidence that a variant of the anaerobic metabolism transcription factor FNR, unique to and isolated from population IR-3-20, plays a role in IR resistance. The F186I allele of FNR exhibits a diminished ability to activate transcription from FNR-activatable promoters, and furthermore reduces levels of intracellular ROS. The FNR F186I variant is apparently capable of enhancing resistance to IR under chronic irradiation conditions, but does not increase cell survival when exposed to acute irradiation. Our results underline the importance of dose rate on cell survival of IR exposure.

## Introduction

Bacterial species that do not display unusual levels of resistance to ionizing radiation can acquire such resistance by directed evolution [1–4]. However, a lack of advanced DNA sequencing technology prevented molecular characterization of evolved IR resistance in studies carried out in the previous fifty years. Over the past decade, we have generated IR resistance in the model bacterium *Escherichia coli* via directed evolution. Modern genomic sequencing methods have facilitated characterization of the evolved populations. We previously subjected four separate populations of *E*. *coli* to 20 cycles of ^60^Co irradiation (sufficient to kill up to 99.9% of the cells) followed in each cycle by survivor outgrowth. The 20 cycles resulted in large gains in IR resistance in all four populations, designated IR-1-20, IR-2-20, IR-3-20, and IR-4-20 [5, 6].

An isolate from population IR-2-20, CB2000, was previously characterized to identify the genetic alterations underlying the IR resistance phenotype [6]. The effort focused on mutations that were fixed in the population and affected genes or pathways altered in additional populations. Though seven mutations made at least a minor contribution, three mutations affecting DNA metabolism accounted for the majority of the IR resistance phenotype of CB2000. These were variants of (a) the DNA repair protein RecA (D276N), (b) the replicative helicase DnaB (P80H), and (c) the putative helicase YfjK (A152D) [6]. Reliance of CB2000 on three variant DNA metabolism proteins for IR resistance implicated a role for enhanced DNA repair in IR resistance. Indeed, biochemical characterization of the RecA D276N variant revealed novel activities consistent with repair of genomes fragmented by IR exposure [7].

Sequencing of isolates from each population revealed a striking trend: RecA, DnaB, and YfjK variants appeared in populations IR-1-20, IR-2-20, and IR-4-20 [5, 6]. This result suggested that, in addition to IR-2-20, both IR-1-20 and IR-4-20 rely at least in part on enhanced DNA repair for IR resistance. However, of seven isolates sequenced from population IR-3-20, none contained mutations in *recA*, *dnaB*, or *yfjK*. Protein variants common to IR-3-20 which appear in the same protein or pathway in at least one other population include those involved in DNA replication restart (PriA V554I), amelioration of reactive oxygen species (ROS) (GsiB L289P and RsxD V45A), cell wall metabolism (NanT F406S), and anaerobic metabolism (FNR F186I) [5, 6]. IR-3-20 also contains a unique intergenic SNP between the *clpP* and *clpX* genes (*clpP*/*clpX* int). Variants of the ClpP and ClpX proteins were also identified in population IR-1-20 [5, 6]. Of these pathways, only protein variants affecting ROS amelioration and cell wall metabolism have been previously associated with minor contributions to IR resistance [6]. Previous studies have suggested that extraordinary ROS amelioration play a major role in IR-resistance of the radioresistant bacterium *Deinococcus radiodurans* [8–11], suggesting that IR-3-20 may have evolved to ameliorate ROS produced by IR rather than enhance existing DNA repair pathways.

A variant of the anaerobic metabolism transcription factor, FNR, is unique to population IR-3-20, although a truncated FNR variant appears in an isolate from IR-1-20 further evolved for another 20 rounds of selection [6]. We now present evidence that altered global transcription through the FNR variant of IR-3-20, F186I, can make a substantial contribution to experimentally-evolved IR resistance, potentially due to an enhancement of ROS amelioration.

## Results

### The FNR F186I variant enhances resistance to γ-ray IR

**Fig 1:**
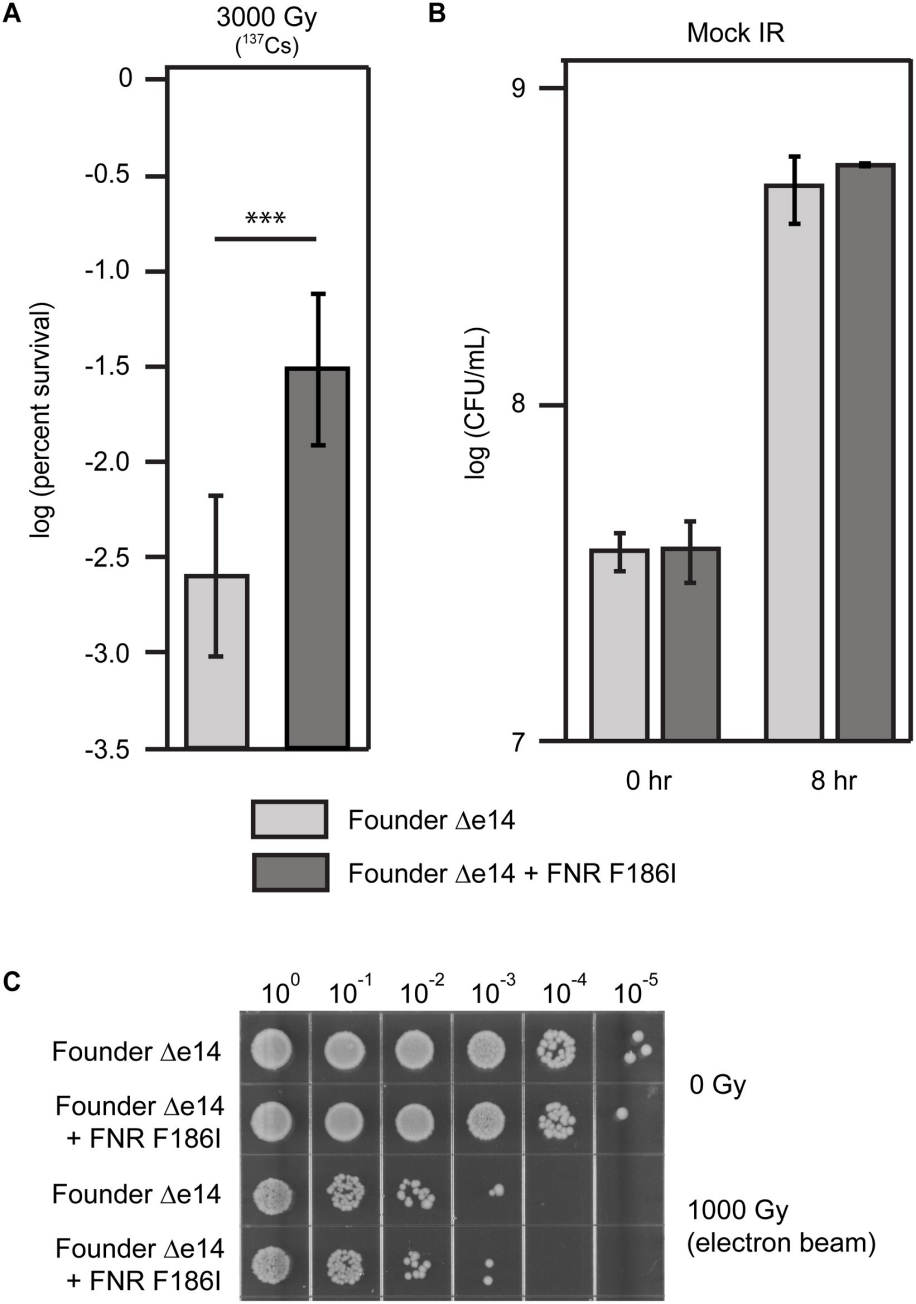
The FNR F186I variant increases resistance to γ-ray IR. **A)** The FNR F186I allele increases survival of cells exposed to γ-ray IR at a dose rate of ~ 6.5 Gy/min. The Y axis represents percent survival. Strains were assayed for survival of exposure to 3,000 Gy at exponential phase growth as described in the Materials and Methods section. The FNR F186I variant was moved from the IR-resistant isolate CB3000 into the Founder Δe14 background as previously described [6, 12]. The results represented indicate the average percent survival of 54 (Founder Δe14) and 23 (Founder Δe14 + FNR F186I) biological replicates. Error bars represent the standard deviation. The difference between the average of each strain is significantly different (p-value < 10^−12^) as calculated with a two-tailed Student’s t-test, as indicated by the ‘***’ symbol. Raw data for Fig 1A is contained in Supporting Information (S1 File. IR resistance assays survival data). **B)** The FNR F186I allele does not increase CFU/mL in mock-IR conditions. Cells were prepared and incubated at room temperature for 8 hours, but not exposed to IR. There is no significant difference (p-value > 0.05) between CFU/mL values for cultures with the wild-type or F186I FNR alleles. The results indicate the average CFU/mL of cultures grown to early exponential phase (OD600 = 0.2), which is the 0 hour time point, and then the same cultures incubated under mock-IR conditions for 8 hours. The experiment was performed in biological triplicate. Raw data for Fig 1B is contained in Supporting Information (S2 File. CFU counts during mock IR). **C)** The FNR F186I allele does not increase IR resistance to high-dose rate electron beam IR delivered at 71 Gy/min. These data represent a single, representative experiment repeated independently. Irradiation with the linear accelerator was carried out as described in the Materials and Methods.

The F186I variant of FNR is the only variant of this protein detected in any of the four populations of *E*. *coli* exposed to 20 iterative cycles of IR selection via ^60^Co irradiation [5, 6]. Another FNR variant (an introduced stop codon at M157) appeared in a population derived from the evolved isolate CB1000, after twenty further cycles of selection [6]. To characterize the impact of the F186I variant, we constructed a strain in which this variant was placed in an otherwise wild type genetic background. As the e14 prophage was lost in all four populations very early in the directed evolution trial [5], we also deleted the e14 prophage to mimic the genetic background in which the FNR F186I mutation arose (we refer to this background as Founder Δe14). We have previously shown that loss of the e14 prophage increases IR resistance, through an unknown mechanism [5]. At a dose of 3000 Gy, administered by a ^137^Cs irradiator at a dose of ~6.5 Gy/min, the FNR F186I variant increases IR resistance relative to Founder Δe14 by approximately 10-fold (Fig 1A). This increase is comparable to that of the major IR resistance-enhancing single mutations from the IR-resistant isolate CB2000 [6], utilizing the same irradiation conditions. This increase in IR resistance is not due to enhanced growth during the long duration of irradiation conditions (1 mL of culture in sealed 1.5 mL tubes incubated at room temperature for 8 hours). In these conditions, without irradiation, there is no significant difference in CFU/mL values of strains with the wild-type or F186I FNR alleles (Fig 1B).

Furthermore, we demonstrate that the IR resistance effects of FNR F186I depended upon the relatively slow irradiation conditions compelled by use of the ^137^Cs irradiator. The dose rate of the ^137^Cs irradiator used for our previous experiments (~6.5 Gy/min) necessitated approximately 8 hours to deliver a dose of 3000 Gy. To shorten the duration of irradiation but maintain equivalent cell killing, we utilized a clinical linear accelerator (Linac) which produces high-energy electron beam IR at a dose rate of ~71 Gy/min. At a dose of 1000 Gy (which kills a similar percentage of Founder Δe14 culture as a dose of 3000 Gy of γ-ray IR) there is no noticeable increase in IR resistance generated by the FNR F186I variant in Founder Δe14 (Fig 1C). These results suggest that this FNR variant confers resistance to a relatively lower dose rate IR over a long duration of exposure (6.5 Gy/min over 8 hours) but not to acute IR exposure (71 Gy/min over 15 min).

### FNR F186I exhibits a diminished capacity to activate transcription

**Fig 2:**
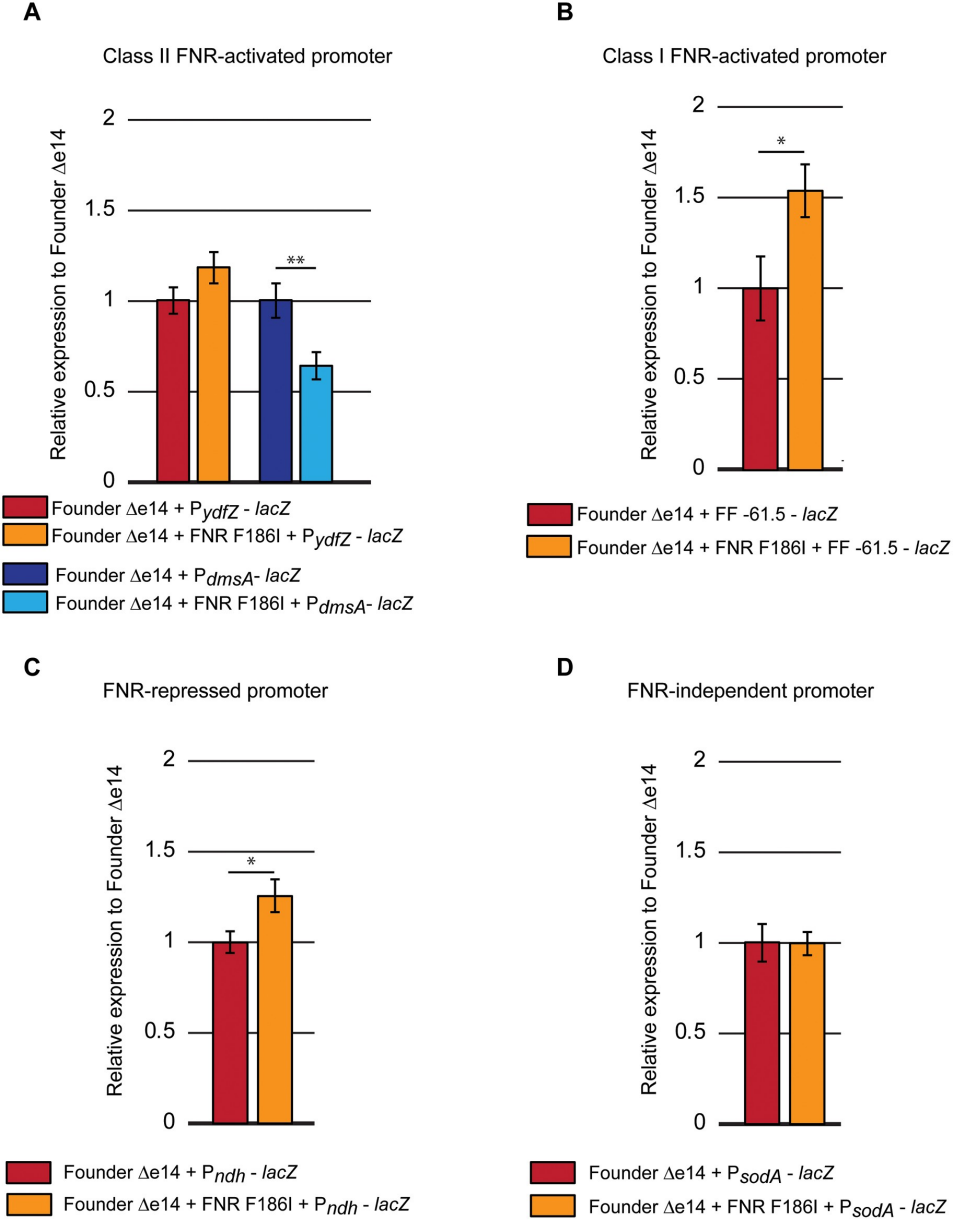
β-galactosidase activity of promoter-*lacZ* fusions in Founder Δe14 + FNR F186I at exponential phase growth. β-galactosidase activity was assayed at exponential phase (OD_600_ = 0.2) β -gal activity was normalized to the average β -gal activity of Founder Δe14 with the appropriate *lacZ* fusion (containing the wild-type FNR F186 allele). These fusions have been previously described [16, 18–20]. The β-galactosidase assay was carried out as described in the Materials in Methods section. These data represent the results of two independent experiments carried out in biological triplicate or duplicate for the repeat experiment. Statistical significance was determined using a two-tailed Student’s t-tests. P-values are indicated using the ‘*’ (p < 0.05), ‘**’ (p < 0.01), and ‘***’ (p < 0.001) symbols. Data for these experiments are contained in the Supporting Information (S3 File. Beta-galactosidase raw data).

We sought to better understand the contribution of the FNR F186I allele to IR resistance. As in previous work, cells were irradiated at a dose rate of ~6.5 Gy/min for over 8 hours to achieve a dose of 3000 Gy [6]. FNR is a Fe-S binding transcription factor that regulates genes related to anaerobic metabolism [13, 14]. It has been previously reported that the F186 residue of FNR contacts the alpha C-terminal domain of RNA polymerase and that variants of residue F186 decrease activation of Class I and Class II FNR-dependent promoters [15, 16]. Class I and Class II promoters are defined by the distance of the FNR dimer binding site from the transcription start site, with FNR binding centered at –61.5 or –71.5 for Class I or –41.5 for Class II [17]. Therefore, we sought to determine the effects, if any, of the F186I variant on FNR-controlled transcription. We utilized a variety of chromosomal transcriptional fusions carrying FNR-dependent and FNR-independent promoters placed upstream of the chromosomal *lacZ* gene (Table 1). The previously described fusions were introduced in Founder Δe14 and Founder Δe14 + FNR F186I. Under routine growth conditions (shaking flasks at 37 °C) and at early exponential phase (OD_600_ = 0.2), we observed no more than small differences (all less than 2-fold) between Founder Δe14 with the wild-type or variant FNR n transcription activation with all promoter fusions (Fig 2).

**Table 1:** Promoter-*lacZ* fusions used in this study.

Promoter	Method of transcriptional control by FNR	Source
*P*_*dmsA*_	Activated; Class II promoter	Elamberg, et al. 2002
P_*ydfZ*_	Activated; Class II promoter	Mettert, et al. 2008
FF -61.5	Activated; Class I promoter	Weber, et al. 2005
P_*ndh*_	Repressed	Elamberg, et al. 2002
P_*sodA*_	Independent	Giel, et al. 2006

These promoter-fusions utilize the endogenous promoter for the *dmsA*, *ydfZ*, *ndh*, and *sodA* genes. The FF -61.5 promoter is a synthetic promoter, where the FNR dimer binding site is placed at the -61.5 position relative to the transcription start site of the fusion.

**Fig 3:**
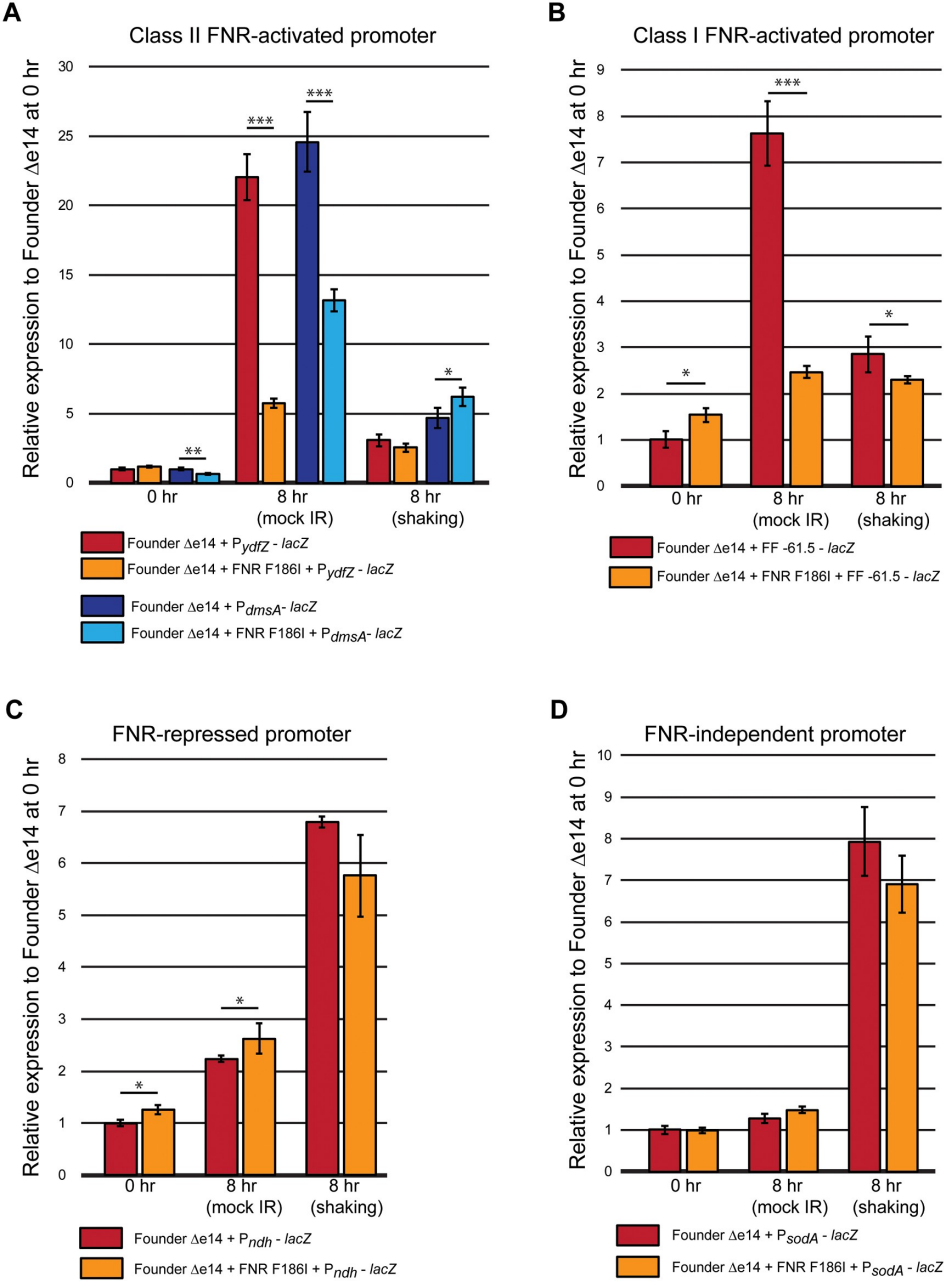
β-galactosidase activity of promoter-*lacZ* fusions in Founder Δe14 + FNR F186I. β-galactosidase activity was assayed in conditions mimicking irradiation assays (exponential phase cells aliquoted in a 1.5 mL tube and incubated at room temperature for 8 hr) with and without shaking. β -gal activity was normalized to the average β -gal activity of Founder Δe14 with the appropriate *lacZ* fusion (containing the wild-type FNR F186I allele) at the 0 hr time point (prior to start 8 hr incubations). These fusions have been previously described [16, 18–20]. The β-galactosidase assay was carried out as described in the Materials in Methods section. These data represent the results of two independent experiments carried out in biological triplicate or duplicate for the repeat experiment. Statistical significance was determined using a two-tailed Student’s t-test. P-values are indicated using the ‘*’ (p < 0.05), ‘**’ (p < 0.01), and ‘***’ (p < 0.001) symbols. Data for these experiments are contained in the Supporting Information (S3 File. Beta-galactosidase raw data).

The bacterial cultures that we routinely irradiated were harvested from early exponential phase culture (OD_600_ = 0.2) (6). Thus, in principle, any effects of the FNR variant should surface under these conditions. However, the dose-rate of the ^137^Cs irradiator and the long duration (~8 hours) of irradiation necessary to delivery 3000 Gy could create additional cellular stresses, such as the reduced oxygen (microaerophilic) conditions necessary to induce FNR activity. A microaerophilic environment should increase the amount of active FNR dimers, which are destroyed by O_2_, and increase expression of the FNR regulon [21]. Therefore, we investigated the effect of a mock-IR condition (defined as 1 mL of culture aliquoted in a sealed 1.5 mL tube, placed on its lid and incubated at room temperature for 8 hours) on expression from FNR-activated promoters. After the mock-irradiation, both Class I and Class II FNR-activated promoters showed increased transcription activation with wild-type FNR relative to that observed in the 0 hr (before the 8 hr incubation was started) time point (Fig 3). The two Class II FNR promoters tested, P_*ydfZ*_ and P_*dmsA*_, had approximately 20-fold increased transcription activation in the mock-IR condition with the wild-type FNR. The observed increase was less but still substantial for the synthetic Class I promoter, FF –61.5, approximately 8-fold. This result suggested that the mock IR condition was indeed producing a microaerophilic environment.

We then went on to test the effects of the FNR F186I variant on the observed transcription activation (Fig 3). In the mock-IR condition, the FNR F186I variant exhibited reduced transcription activation from all FNR-activatable promoters (a reduction of approximately 2- to 5-fold compared to the wild-type FNR allele for each respective promoter). The effects of FNR F186I appeared to be focused only on the FNR-activatable promoters. Strains with either the wild-type FNR or F186I variant showed no difference greater than 2-fold in expression from the FNR-repressed P_*ndh*_ or the FNR-independent P_*sodA*_ promoters under the mock-IR condition.

The FNR F186I allele thus appears to reduce the transcriptional activation of FNR-activatable promoters under conditions of reduced oxygen. To confirm that the effects of FNR F186I were specific to microaerophilic conditions, we tested the effects of the variant under aeration. Strains with each promoter-fusion were subjected to rocking for the same duration and temperature as the mock-IR experiments. For all FNR-activatable promoters, transcription decreased in aerated conditions compared to the microaerophilic mock-IR conditions (Fig 3). Increased transcription from the FNR-repressed P_*ndh*_ fusion in both strains under aerated conditions further suggests low levels of active FNR dimers in this condition. In addition, increased transcription from the FNR-independent P_*sodA*_ promoter, *sodA* encoding the Mn-binding superoxide dismutase of *E*. *coli*, for both strains in aerated conditions further indicates the aerobic nature of the shaking environment, as superoxide is generated by aerobic respiration [22]. These results indicate that the FNR F186I variant decreases transcription from FNR-activatable promoters under the microaerophilic conditions of our irradiation trials.

### FNR F186I reduces ROS in shaking culture

**Fig 4:**
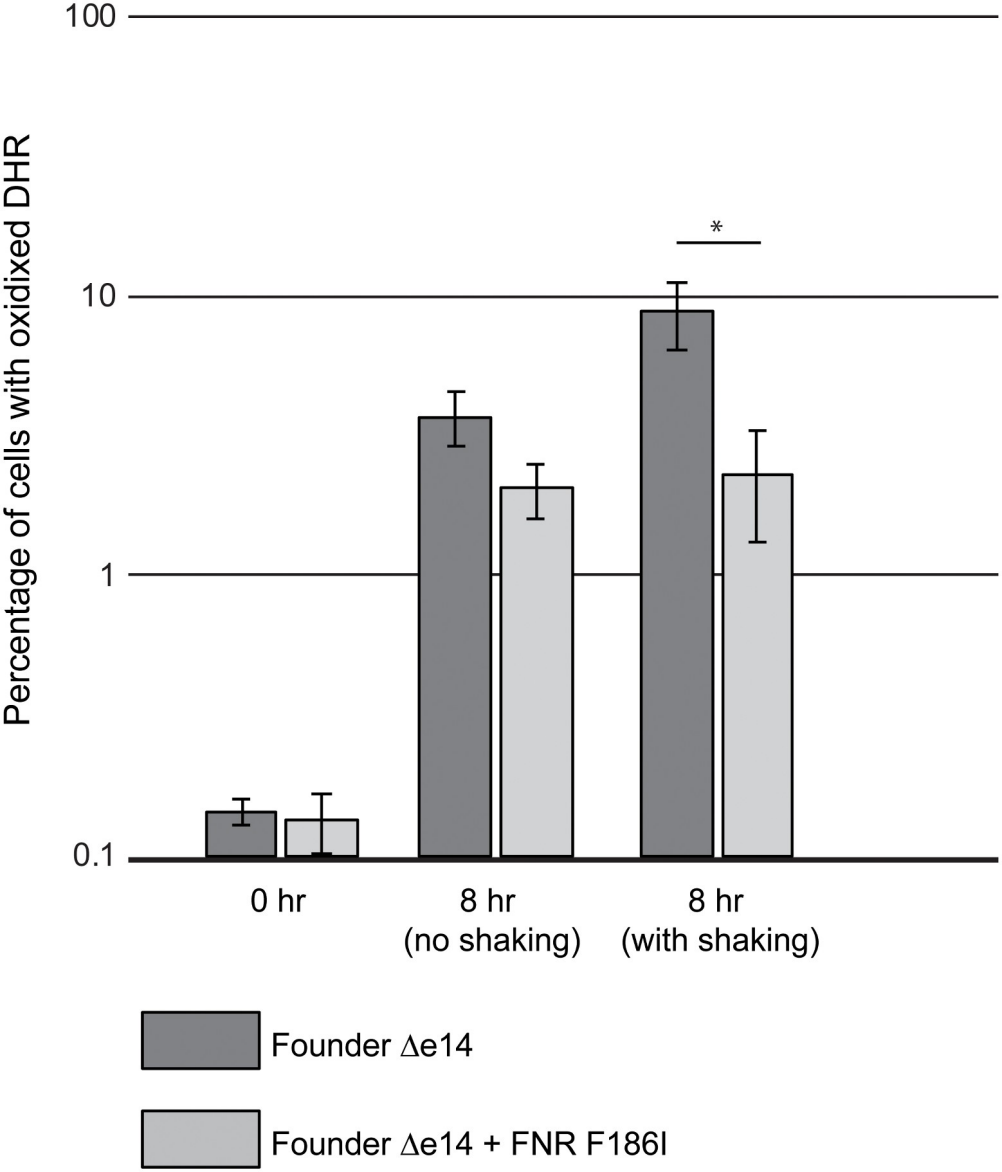
The FNR F186I reduces oxidation of the fluorescent dye dihydrorhodamine-1,2,3. Founder Δe14 + FNR F186I has reduced levels of oxidized DHR in mock-IR conditions with and without shaking compared to Founder Δe14. These data represent a single, representative experiment conducted with biological triplicate of each strain. Experiments were carried out as described in the Materials and Methods. Statistical significance was determined using a two-tailed Student’s t-test. P-values are indicated using the ‘*’ (p < 0.05) symbol. Data for these experiments are contained in the Supporting Information (S4 File. Oxidized DHR data).

There is no direct link of the FNR regulon to DNA repair, so enhanced DNA repair was unlikely to explain the effects of FNR F186I. A potential mechanism for IR-resistance conferred by a defective FNR variant is amelioration of IR-generated ROS. To quantify intracellular ROS, we used dihydrorhodamine-1,2,3 (DHR), a dye which fluoresces once oxidized. DHR was added to culture incubated under the mock irradiation condition, or to control cultures subjected to shaking at the same temperature as carried out for the β-galactosidase assays. Under the mock-IR condition (no shaking), the difference in the level of detectable oxidation between strains with the wild-type or the F186I variant FNR alleles was not statistically significant (Fig 4). However, after 8 hours of incubation with shaking, Founder Δe14 with the FNR F186I variant exhibited detectable ROS that were reduced by approximately 4-fold relative to Founder Δe14 with the wild-type FNR allele. Furthermore, Founder Δe14 with wild-type FNR allele showed increased ROS in shaking versus stationary conditions, whereas Founder Δe14 with the FNR F186I variant shows similar levels of ROS with or without shaking. These results suggest that the FNR F186I allele may somehow enhance ROS amelioration. As the main effects of F186I on transcription of FNR-activatable promoters appears to occur under the mock-IR (microaerophilic) condition, the molecular origin of the F186I effect on ROS generation is not yet evident.

### FNR F186I enhances growth in rich medium without selection

**Fig 5:**
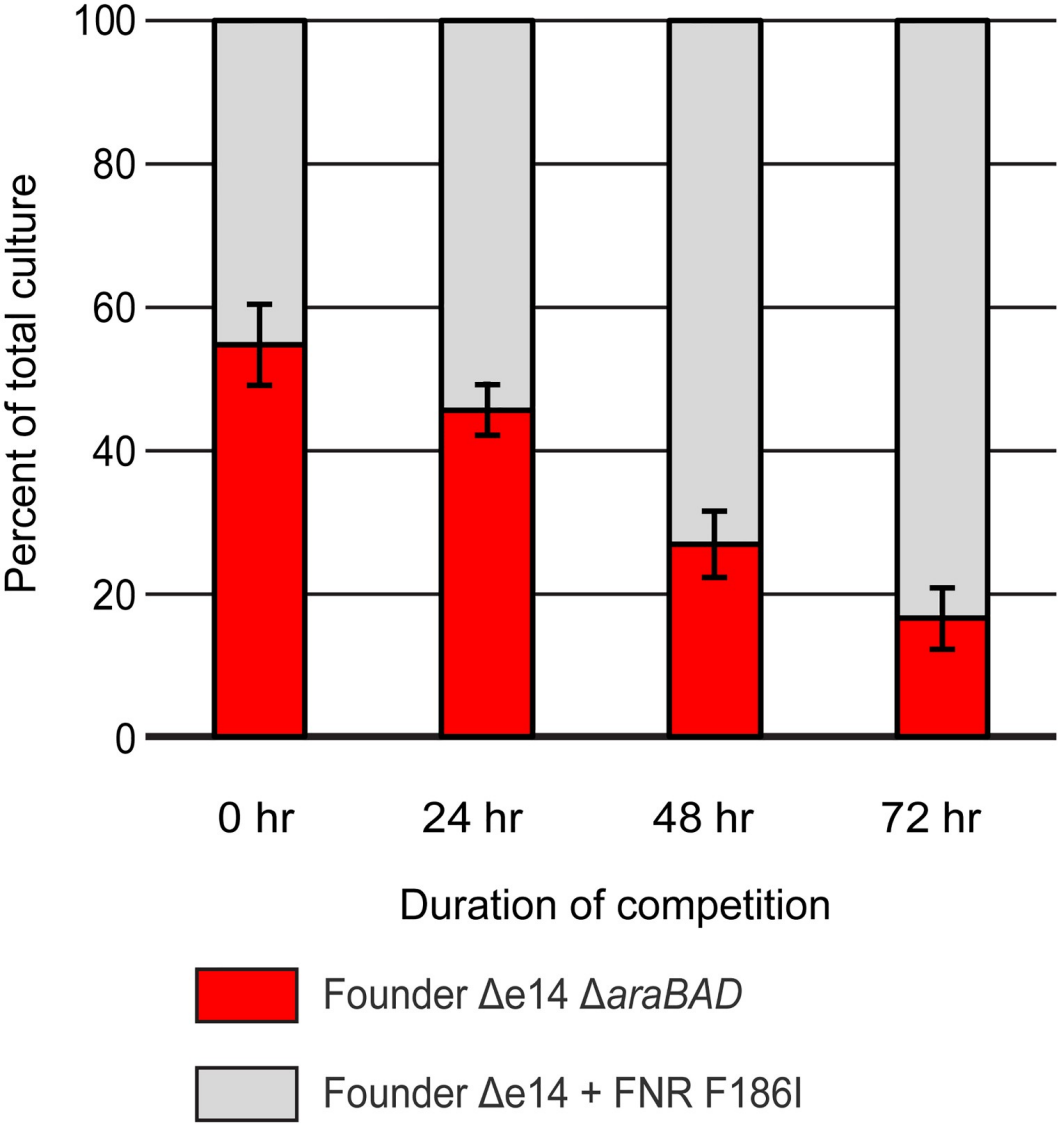
FNR F186I enhances growth competition of Founder Δe14 in mixed culture without selection. Founder Δe14 + FNR F186I outcompetes Founder Δe14. These data represent a single, representative competition against Founder Δe14 without the *araBAD* operon (a neutral mutation used to differentiate strains in the competition; loss of the *araBAD* operon results in red colonies on TA medium). Growth competitions were carried out as described in the Materials and Methods. Data for these experiments are contained in the Supporting Information (S5 File. Growth competition raw data).

The directed evolution protocol used to generate highly IR-resistant *E*. *coli* has an inadvertent secondary selection step for enhanced growth during the outgrowth of irradiated survivors [5, 6]. To determine if the FNR F186I allele had any beneficial effect on growth, we utilized a previously described growth competition protocol in the absence of IR selection. The growth competition involves mixing the two bacterial cultures to be compared in approximately 50:50 ratios. The two are then grown together in fresh medium over 24 hour periods. One is distinguished from the other by the presence of a neutral mutation (deletion of the *araBAD* operon, which confers a red color to colonies when grown on tetrazolium arabinose (TA) indicator plates) to permit color-based scoring of mixed populations [6, 23]. After a duration of 48 hours, the growth competition assay revealed enhanced growth by Founder Δe14 with the FNR F186I allele compared to the parent strain (Fig 5). This is the first evidence that mutations selected for during our irradiation trials can both increase IR resistance as well as enhance growth without IR selection.

## Discussion

Utilizing four experimentally evolved populations of IR-resistant *E*. *coli* (IR-1-20, IR-2-20, IR-3-20, and IR-4-20) [5], we have begun to elucidate the means by which a naturally IR-sensitive organism can withstand extreme doses of IR. We have previously described experimentally-evolved IR resistance through enhanced DNA repair (the major contributors being RecA D276N, DnaB P80H, and YfjK A152D) [6]. Enhanced DNA repair appears to be a major mechanism of IR resistance in three of the four evolved populations, as IR-1-20, IR-2-20, and IR-4-20 each have prominent mutations affecting the RecA, DnaB, and YfjK proteins. IR-3-20 is unique in its lack of variants of these proteins, and the presence of an FNR variant. Here we demonstrate that this FNR variant (F186I) makes a significant contribution to IR resistance (Fig 1A). Interestingly, the F186I allele of FNR appears to only enhance resistance to IR delivered over a long duration of irradiation, as high dose rate IR (71 Gy/min of a high-energy electron beam versus 6.5 Gy/min of γ-rays) kills a similar percentage of strains with the wild-type or F186I variant alleles (Fig 1C). Further, FNR F185I reduces activation of FNR-activatable promoters under conditions of reduced oxygen (Fig 3). The FNR F186I variant also reduces ROS generation under conditions of vigorous aeration (Fig 4). In general, the FNR F186I allele appears to increase IR resistance through a much different mechanism than the previously described DNA repair variants. [5, 6]. That mechanism may involve, at least in part, enhanced amelioration of ROS, a mechanism prominently advanced by research into the highly radiation resistant bacterium *Deinococcus radiodurans* by Daly and colleagues (8–11) Finally, we present the first evidence that mutations which enhance IR resistance may also enhance growth in mixed culture without selection by IR (Fig 5), indicating that selection cycles for IR-resistance also yielded selection for growth.

We observed that the FNR F186I variant exhibits a decreased capacity to activate FNR-activatable promoters (Fig 3). This phenotype was specific to culture in a mock-IR condition, where a microaerophilic environment was created by the duration of the assay and stationary placement of the culture tube (Figs 2 and 3). Although we have shown that the F186I allele is deficient in promoter activation, the downstream effects of the FNR F186I variant that lead to IR resistance are unknown. While previously described mutations that enhance IR resistance are related to DNA repair [5, 6], the FNR regulon does not include known DNA repair proteins. Microarray analysis has implicated FNR as a regulator of 103 genes related to anaerobiosis, activating 68 and repressing 35 [24]. Of these FNR-controlled genes, reducing expression of the *fnrS* small regulatory RNA is the most obvious candidate for increasing IR resistance. The *fnrS* regulatory RNA is known to repress translation of the Fe-binding superoxide dismutase, SodB, and the transcription factor MarA, which activates transcription of the Mn-binding superoxide dismutase, SodA [25, 26]. Reducing levels of *fnrS* may increase translation of SodA and SodB, therefore leading to enhanced ROS amelioration and IR resistance. This hypothesis is consistent with decreased oxidation of the DHR dye in Founder Δe14 with the FNR F186I variant compared to the wild-type allele (Fig 4). However, the reduction in ROS is not evident under the same mock IR conditions where the transcriptional effects of FNR F186I are prominent, so a full explanation of the contribution of this FNR allele to IR resistance may lie elsewhere. The FNR regulon includes 11 genes and four operons of unknown function for which transcription is activated by FNR. It is possible that one or more of these genes may play a role in the observed increase in IR resistance contributed by FNR F186I [27]. It has previously been observed that in *D*. *radiodurans*, exposure to IR will induce altered gene expression to reduce metabolic ROS [28].

Interestingly, the FNR F186I variant only appears to enhance IR resistance when dose is delivered at a slow rate over a long duration. This variant can increase survival by approximately 10-fold at a dose of 3000 Gy delivered at 6.5 Gy/min, but has no effect on survival when a dose of 1000 Gy is delivered at 71 Gy/min (this dose kills an approximately equivalent percentage of Founder Δe14 when 3000 Gy is delivered at either 6.5 or 71 Gy/min) (Fig 1C). These results suggest that the FNR F186I allele was selected for at least in part by the long duration of irradiation, and that this variant is an adaptation for survival of chronic, and not acute, IR exposure. This phenotype suggests the importance of how quickly damage accumulates in chronic versus acute irradiation conditions, and that cells may be able to survive damage spread over a long duration but not severe damage over a short period of time. Previous work in Eukaryotic model systems have indicated the importance of dose rate on cell survival and suggested that higher dose rates inflict more severe DNA damage and greater cell death [29–31].

The differences in cell survival between irradiation conditions noted in this study are of particular importance in the current state of radiobiology research. A new project administered by the Office of Radiological Security (United States of America Department of Energy), the Cesium Irradiator Replacement Project (CIRP), has initiated decommissioning of radioisotope sources of irradiation at academic institutions in favor of electronic X-ray or electron-beam sources. As this transition progresses, much work will need to be carried out to characterize the biological effects of changing irradiation conditions and dose rates in order to adequately compare past, in progress, and future research.

A surprising phenotype conferred by the FNR F186I variant is the ability to outcompete the parent strain with wild-type FNR in direct competition without IR selection. The outgrowth step organic to the experimental evolution protocol provides a chance to select for mutations that enhance growth in the absence of IR. The FNR F186I variant appears to not only increase IR resistance, but also fitness in rich media. It is not clear how altered transcription within the complex FNR regulon could affect growth in aerated, rich medium. Although FNR dimers are inactivated by the presence of oxygen, our data presented here show as much as 5-fold increased transcription from FNR-controlled promoters in aerated culture (Fig 3), suggesting there is a small pool of active FNR even in aerobic environments. Activation of FNR-controlled promoters in aerobic conditions has been previously been noted in similar experiments [19]. It may be that proteins involved in anaerobic metabolism that are regulated by FNR can interfere with aerobic metabolism, even at low levels. Decreasing expression of these proteins due to the FNR F186I variant may reduce competition between aerobic and anaerobic metabolism, allowing for enhanced growth in aerobic environments.

Altered central metabolism through the FNR regulon appears to be a new potential pathway to IR resistance in *E*. *coli* under at least some conditions of irradiation. Further experimental evolution studies may elucidate the relationship between enhanced DNA repair and altered central metabolism, as these pathways may (or may not) appear within the same evolving populations over time.

## Materials and methods

### Growth conditions and bacterial strains used in this study

Unless otherwise stated, *E*. *coli* cultures were grown in Luria-Bertani (LB) broth [32] at 37°C with aeration. Overnight cultures were grown in a volume of 3 mL for 16 to 18 hr. Exponential phase cultures were routinely diluted 1:100 in 10 mL of LB medium in a 50 mL Erlenmeyer flask and were grown at 37°C with shaking at 200 rpm and were harvested at an OD_600_ of 0.2, unless otherwise noted. After growth to an OD_600_ of 0.2, cultures were placed on ice for 10 min to stop growth before being used for assays.

*E*. *coli* were plated on 1.5% LB agar medium [32] and incubated at 37°C. Cultures were plated on tetrazolium agar (TA) for growth competition assays when noted [23].

All strains used for *in vivo* assays in this study are mutants of *E*. *coli* K-12 derivative MG1655 [33]. Strains are listed in the Supporting Information (S1 Table. Strains used in this study). Genetic manipulations to transfer mutations or delete genes were carried out as previously described [34, 35]. Promoter fusions to *lacZ* were transferred via P1 transductions from the parent strains and recombination of the promoter-*lacZ* fusion at the chromosomal *lacZ* locus was confirmed via colony PCR as previously described [36].

### Serial dilutions and CFU/mL determination

All serial dilutions were carried out in 1X phosphate-buffered saline (PBS) (for 1 L: 8 g NaCl, 0.2 g KCl, 1.44 g Na_2_HPO_4_, KH_2_PO_4_ 0.24 g with 800 mL dH_2_O, adjust pH with HCl to 7.4, then add remaining 200 mL dH_2_O). Unless otherwise stated, serial dilutions were carried out with serial 1:10 dilutions of 100 μL of culture or previous dilution into 900 μL 1X PBS. Before transfer to the next dilution tube, samples were vortexed for 2 seconds and mixed by pipetting to ensure mixing. 100 μL of appropriate dilutions were aliquoted onto agar plates of the appropriate medium and were spread-plated utilizing an ethanol-sterilized, bent glass rod. For spot plating, 10 μL of each dilution was aliquoted onto agar plates of the appropriate medium and spots were allowed to dry before plates were incubated as in *Growth conditions*.

CFU/mL was calculated using the highest CFU count for each strain assayed that remained between 30 and 300 CFU (ex: 250 CFU on a 10^−4^ dilution plate would be used for calculation over 40 CFU on a 10^−5^ dilution plate).

### Ionizing radiation resistance assay with ^137^Cs irradiator

Cells from a single colony of each strain were cultured overnight and then grown to an OD_600_ of ~ 0.2 as in Growth conditions. One mL aliquots in 1.8 mL Eppendorf tubes were mixed by vortexing for 2 s and a 100 μL aliquot was removed and added to 900 μL PBS on ice as an initial 1:10 dilution for the non-irradiated control. Undiluted samples were then irradiated in a Mark I ^137^Cs irradiator (J. L. Shepherd and Associates) for a time corresponding to 3 kGy (~ 6.5 Gy/min). Irradiated samples as well as the non-irradiated samples were serial diluted and plated using typical methods. CFU/mL was determined for irradiated and un-irradiated samples, and CFU/mL was determined. Initial cell densities ranged from 1–6 x 10^7^ CFU/ml. For each experiment, each individual strain was tested in biological triplicate.

### β-galactosidase assay

The β-galactosidase assay was adapted from a previously described protocol [32]. Cultures to be assayed were prepared by incubating cells from a single colony of each strain overnight at 37 °C with aeration. This resulting overnight culture (grown 15–18 hr) was treated as the stationary phase culture to be assayed. Exponential phase cultures were grown by inoculating 10 mL of LB broth in a 50 mL Erlenmeyer flask with 70 μL of overnight culture and grown at 37 °C with shaking to an OD_600_ 0.2. These cultures were placed on ice for at least 5 minutes to stop growth before use. To perform a mock irradiation, two separate aliquots of 900 μL of exponential phase cultures in 1.5 mL Eppendorf tubes were inverted and incubated in the dark at room temperature (~24 °C) for 8 hr. Two aliquots for each replicate ensures that there is sufficient volume for 1 mL of culture to determine β-galactosidase activity and 100 μL to determine OD_600_.

The β-galactosidase assay was carried out as follows. An OD_600_ reading was taken of each culture, and an appropriate amount (1 mL for exponential phase and mock irradiation cultures, and 50 μL for stationary phase) was aliquoted into 2 mL microcentrifuge tubes. Cells were pelleted via centrifugation at 6900 xg for 3 min, and supernatant was removed. Cells were resuspended in 1 mL Z buffer (0.06 M Na_2_HPO_4_, 0.04 NaH_2_PO4, 0.01 KCl, 0.001M MgSO_4_, to volume with purified dH_2_O), and 1 mL Z buffer was aliquoted in a 2 mL microcentrifuge tube for a blank sample. One-hundred μL chloroform and 50 μL 0.1% SDS were added to each tube. Each sample was then vortexed for 10 s and incubated at 4 °C for at least 10 min. Three samples at a time were removed from 4 °C and placed in a 28 °C water bath for 5 min. Two-hundred μL 4 mg/mL O-Nitrophenyl β-D-Galactopyranoside (ONPG) (Sigma-Aldrich, St. Louis, MO Cat#: N1127) dissolved in Z buffer was added to each sample. The development of yellow coloration for each sample was timed. Once the sample had become yellow (or after 30 minutes) the reaction was stopped by adding 500 μL 1M Na_2_CO_3_ and samples were placed on ice. All samples were spun for 10 min at 17000 xg in a microcentrifuge at 4 °C. One mL was removed from each sample, and the OD_420_ and OD_550_ was read. To determine β-galactosidase activity, the following equation was used where *t* is time of the reaction and *v* is the volume of culture used:

$$Activity=\frac{1000*(\left({OD}_{420}-(1.75*{OD}_{550})\right))}{t*v*{OD}_{600}}$$

All β-galactosidase assays were carried out using biological triplicate. To determine relative β-galactosidase activity compared to the parent strain, the activity of each mutant strain replicate was divided by the average activity of the wild-type triplicate in the given experiment.

### Detection of reactive oxygen species with dihydrorhodamine- 1,2,3

Detection of ROS using the dihydrorhodamine-1,2,3 (Thermo Fisher Cat #: D23806) dye was carried out as previously described [37]. Briefly, 1 mL of cells washed three times with PBS was mixed with 1 mM DHR. This mixture was incubated for 30 minutes at 4°C protected from light. The samples were then run on a BD Accuri C6 Flow Cytometer located in the Biochemistry Instrumentation Facility (UW- Madison) using a FSC-H cutoff of 10,000. DHR fluorescence was detected using excitation and emission wavelengths of 488 nm and 530 nm, respectively.

### Ionizing radiation resistance assay with linear accelerator

Cells from a single colony of each strain were cultured overnight and then grown to an OD_600_ of ~ 0.2 as in Growth conditions. One mL aliquots in 1.8 mL Eppendorf tubes were mixed by vortexing for 2 s and a 100 μL aliquot was removed and added to 900 μL PBS on ice as an initial 1:10 dilution for the non-irradiated control.

Samples were maintained at 4 °C and transported to the University of Wisconsin Medical Radiation Research Center (UWMRRC) Varian 21EX clinical linear accelerator (Linac) facility for irradiation. The total transport time was approximately 15 min to and from the Linac facility. For each irradiation, the Linac was set to deliver a beam of electrons with 6 MeV of energy to uniformly irradiate all samples (a total of 14) at once. To accomplish this, a special high-dose mode called HDTSe¯ was utilized, which resulted in a dose rate to the samples of approximately 71 Gy/min. The sample tubes were placed horizontally and submerged at a depth of 1.3 cm (measured to the center of the tube’s volume) in an ice-water filled plastic tank and set to a source-to-surface distance (SSD) of 61.7 cm. A 30 x 30 cm^2^ square field size was set at the Linac console, which gave an effective field size at this SSD of 18.5 x 18.5 cm^2^. This is ample coverage to provide a uniform dose to all of the sample vials. The monitor unit calculations (determination of the amount of time to leave the Linac on) were based on the American Association of Physicists in Medicine (AAPM) Task Group 51 protocol for reference dosimetry [38]. This is the standard method for determining dose per monitor unit in water for radiation therapy calculations. Once the dose was determined in the AAPM Task Group 51 reference protocol conditions (SSD = 100 cm and depth = 10 cm), an ion chamber and water-equivalent plastic slabs were used to translate this dose to the specific conditions used in this project.

After irradiation, samples were serial diluted and spot plated as described in *Serial dilutions*.

### Growth competition assay

This assay was adapted from a previously published protocol [23]. To differentiate strains within the competition, a fitness-neutral deletion of the *araBAD* operon was introduced into one of the two strains. This deletion results in red colonies on tetrazolium arabinose (TA) agar plates [23]. Briefly, overnight culture of each strain to be competed were mixed 1:1 in a 1.5 mL microcentrifuge tube. Samples were mixed by vortexing for 5 s and were serial diluted 1:10 in 900 μl phosphate-buffered saline (PBS) to a final dilution of 10^−6^. One hundred μL of the final dilution was spread plated onto TA agar plates to assay for CFU. Seventy μl of the remaining cell mixture was used to inoculate 5 mL of fresh LB media for growth overnight. This overnight culture was used to inoculate fresh media the following day, and 100 μl was serially diluted and plated as noted above. This procedure was repeated twice more over a period of two days. The number of white versus red CFU was noted after each day of the competition and the total percentage of the culture for each competitor was determined.

## Supporting information

S1 File IR resistance assays survival data. (XLSX)

S2 File CFU counts during mock IR. (XLSX)

S3 File Beta-galactosidase raw data. (TIF)

S4 File Oxidized DHR data. (XLSX)

S5 File Growth competition raw data. (XLSX)

S1 Table Strains used in this study. (DOCX)
