# Supplementary figures and images for: A variant of the Escherichia coli anaerobic transcription factor FNR exhibiting diminished promoter activation function enhances ionizing radiation resistance
Source: PLoS One. 2019 Jan 23;14(1):e0199482. doi: 10.1371/journal.pone.0199482 (PMC6343905; doi:10.1371/journal.pone.0199482)

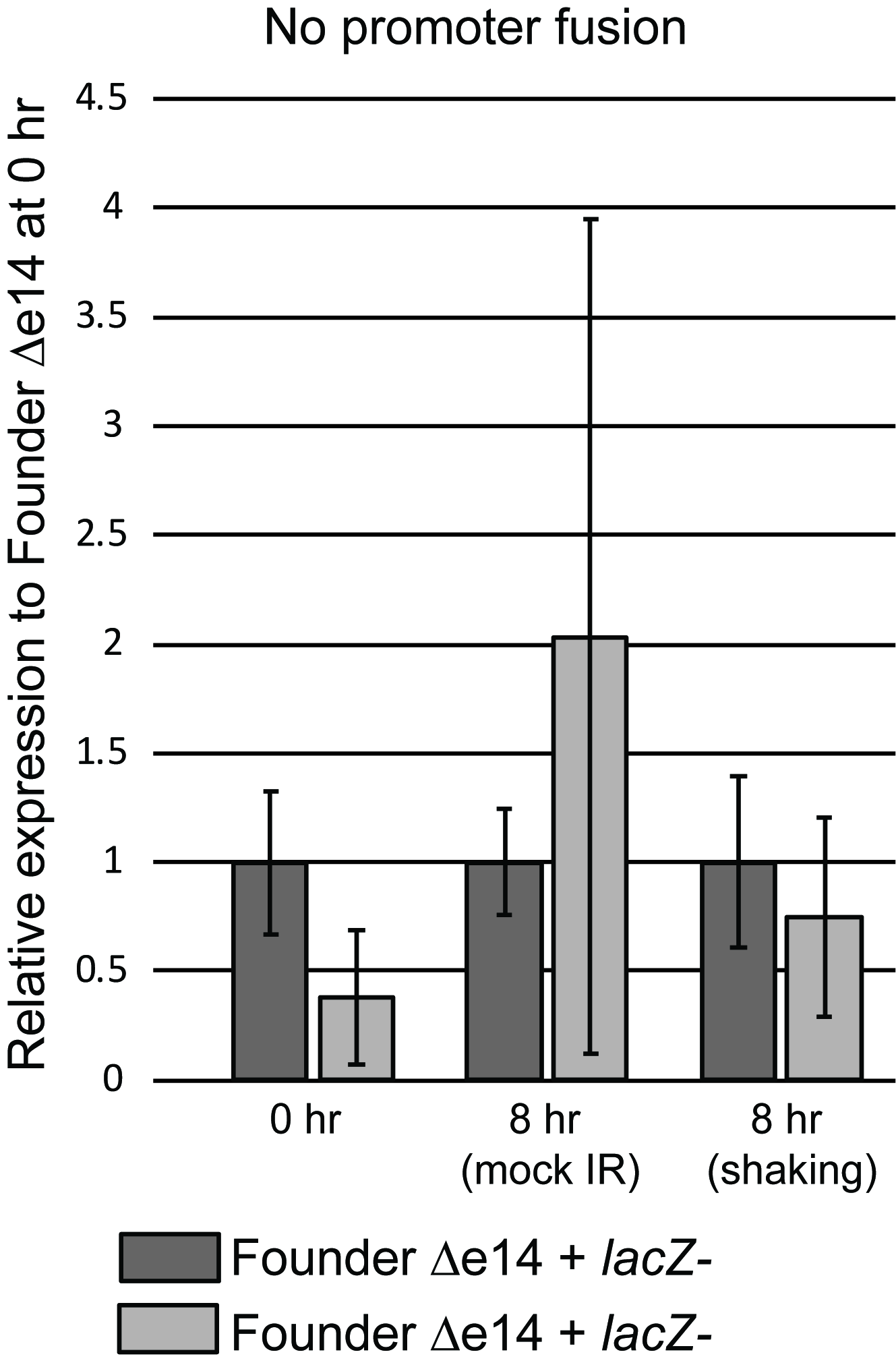

Supplement: S3 File — (TIF) [file pone.0199482.s003.tif]
