# Supplementary material for: A variant of the Escherichia coli anaerobic transcription factor FNR exhibiting diminished promoter activation function enhances ionizing radiation resistance
Source: PLoS One. 2019 Jan 23;14(1):e0199482. doi: 10.1371/journal.pone.0199482 (PMC6343905; doi:10.1371/journal.pone.0199482)
Supplement: S1 Table — (DOCX) [file pone.0199482.s006.docx]

**S1 Table. Strains used in this study.**

| **Strain** | **Relevant Genotype** | **Source** |
| --- | --- | --- |
| MG1655 | YbhJ L54I + MntP G25D + RIP321 A 4296380* ACG + GlpR C 3560455* CG + GatC ACC 2173360* A | Blattner, et al. 1997 |
| EAW7704 (Founder Δe14)­ | MG1655 Δe14 + RbsR L92R + CytR Q110 stop + *fabI*/*ycjD* int (G 1351174* A) + *yifN*/*ppiC* int (T 3959934* C) | Harris, et al. 2009 |
| EAW354 | Founder Δe14 + FNR F186I | This study |
| STB75 | Founder Δe14 Δ*araBAD* | This study |
| PK3292 | P*_dmsA_* - *lacZ* | Elamberg, et al. 2002 |
| PK8202 | P*_ydfZ_* - *lacZ* | Mettert, et al. 2008 |
| PK3286 | P*_ndh_* - *lacZ* | Elamberg, et al. 2002 |
| PK7564 | P*_sodA_* - *lacZ* | Giel, et al. 2006 |
| PK7028 | FF -61.5 - *lacZ* | Weber, et al. 2005 |
| STB107 | FounderΔe14 Δ*lacIZYA* | This study |
| STB127 | FounderΔe14 + FNR F186I Δ*lacIZYA* | This study |
| STB156 | Founder Δe14 + P*_ydfZ_ - lacZ* | This study |
| STB157 | Founder Δe14 + P*_dmsA_ - lacZ* | This study |
| STB166 | Founder Δe14 + FF -61.5 - *lacZ* | This study |
| STB159 | Founder Δe14 + P*_sodA_ - lacZ* | This study |
| STB160 | Founder Δe14 + P*_ndh_ - lacZ* | This study |
| STB161 | Founder Δe14 + FNR F186I + P*_ydfZ_ - lacZ* | This study |
| STB162 | Founder Δe14 + FNR F186I + P*_dmsA_ - lacZ* | This study |
| STB167 | Founder Δe14 + FNR F186I + FF -61.5 - *lacZ* | This study |
| STB164 | Founder Δe14 + FNR F186I + P*_sodA_ - lacZ* | This study |
| STB165 | Founder Δe14 + FNR F186I + P*_ndh_ - lacZ* | This study |

Strains generated in ‘This study’ were constructed as described in the Materials and Methods. The ‘*’ symbol indicates that the nucleotide position refers to the NCBI GenBank U00096.3 reference sequence.
